# Predicting Continental Scale Malaria With Land Surface Water Predictors Based on Malaria Dispersal Mechanisms and High‐Resolution Earth Observation Data

**DOI:** 10.1029/2023GH000811

**Published:** 2023-10-10

**Authors:** Maurice W. M. L. Kalthof, Mathieu Gravey, Flore Wijnands, Derek Karssenberg

**Affiliations:** ^1^ Institute for Environmental Studies (IVM) Vrije Universiteit Amsterdam Amsterdam The Netherlands; ^2^ Department of Physical Geography Utrecht University Utrecht The Netherlands; ^3^ Institute for Interdisciplinary Mountain Research Österreichische Akademie der Wissenschaften Innsbruck Austria; ^4^ Institutionen för Geologiska Vetenskaper Stockholm University Stockholm Sweden

**Keywords:** malaria, surface water, machine learning, remote sensing, *Plasmodium falciparum*, malaria prediction

## Abstract

Despite malaria prevalence being linked to surface water through vector breeding, spatial malaria predictors representing surface water often predict malaria poorly. Furthermore, precipitation, which precursors surface water, often performs better. Our goal is to determine whether novel surface water exposure indices that take malaria dispersal mechanisms into account, derived from new high‐resolution surface water data, can be stronger predictors of malaria prevalence compared to precipitation. One hundred eighty candidate predictors were created by combining three surface water malaria exposures from high‐accuracy and resolution (5 m resolution, overall accuracy 96%, Kappa Coefficient 0.89, Commission and Omission error 3% and 13%, respectively) water maps of East Africa. Through variable contribution analysis a subset of strong predictors was selected and used as input for Boosted Regression Tree models. We benchmarked the performance and Relative Contribution of this set of novel predictors to models using precipitation instead of surface water predictors, alternative lower resolution predictors, and simpler surface water predictors used in previous studies. The predictive performance of the novel indices rivaled or surpassed that of precipitation predictors. The novel indices substantially improved performance over the identical set of predictors derived from the lower resolution Joint Research Center surface water data set (+10% *R* ^2^, +17% Relative Contribution) and over the set of simpler predictors (+18% *R* ^2^, +30% Relative Contribution). Surface water derived indices can be strong predictors of malaria, if the spatial resolution is sufficiently high to detect small waterbodies and dispersal mechanisms of malaria related to surface water in human and vector water exposure assessment are incorporated.

## Key Points

- Surface water predictors can be equally strong or better predictors for malaria compared to precipitation
- Surface water predictors predict malaria better if derived from higher resolution maps
- Surface water predictors become stronger if they resemble the processes of malaria dispersion more closely

## 1 Introduction

Malaria case incidence has declined globally by 29% between 2000 and 2019 (World Health Organization, 2020) and malaria deaths have reduced from 736,000 in 2000, to an estimated 409,000 deaths in 2019. However, a mere 30 countries account for approximately 95% of malaria cases and deaths, and this group consists nearly entirely of countries in sub‐Saharan Africa (SSA) (World Health Organization, 2020). As the WHO aims to reduce global malaria mortality rates and case incidence by at least 90% by 2030, it is crucial to target malaria in this region.

Malaria incidence is reduced by increasing access to prevention, diagnosis, and treatment (World Health Organization, 2015, 2020). However, resources to fund such interventions are limited and fall short of what is required (Cibulskis et al., 2016; Patouillard et al., 2017). Therefore, targeting available resources in cost‐effective ways at malaria hotspots is necessary to ensure sufficient progress toward global malaria targets (Bousema et al., 2012; World Health Organization, 2020).

To identify the location of such hotspots, statistical models make use of spatial predictor variables to map malaria incidence. In general, the inputs to these models can be divided into two categories (McMahon et al., 2021; Solano‐Villarreal et al., 2019). First, social predictors of malaria transmission, consisting of predictors such as limited access to healthcare (Weiss et al., 2018), population density, and migration (Chuquiyauri et al., 2012; McMahon et al., 2021; Solano‐Villarreal et al., 2019). Second, environmental predictors, which influence the mosquito life cycle, mortality, fecundity as well as the pathogenic development of malaria parasites. Approximately 80%–90% of total malaria cases are estimated to be related to environmental factors (CDC, 2020; Ferrao et al., 2018). The most common environmental predictors are precipitation, surface water, air temperature, and vegetation (McMahon et al., 2021; Sinka et al., 2010; Solano‐Villarreal et al., 2019), of which temperature and precipitation are generally reported as being the most influential (Ferrao et al., 2018; Kleinschmidt et al., 2001; McMahon et al., 2021; Noor et al., 2008; Sinka et al., 2010; Solano‐Villarreal et al., 2019).

When comparing malaria predictor strength, many studies show surface water predictors are performing worse compared to precipitation predictors (Ferrao et al., 2018; Kleinschmidt et al., 2001; McMahon et al., 2021; Noor et al., 2008; Sinka et al., 2010; Solano‐Villarreal et al., 2019). However, aside from affecting relative humidity (Zacarias & Andersson, 2011), precipitation itself does not increase malaria transmission; it leads to pool forming, a factor which is further mediated by the hydrology and geomorphology of an area (Smith et al., 2013). The resultant surface water pools increase vector abundance, and this subsequently leads to higher malaria transmission rates (Ross, 1908; Smith et al., 2013). Therefore, it is surprising that surface water, which has a shorter causal link to malaria, appears to be a weaker statistical predictor of malaria compared to precipitation.

We hypothesize that the limited effectiveness of current studies in predicting malaria prevalence is due to inadequate representation of surface water exposures. This is mainly due to the utilization of coarse‐resolution surface water occurrence maps and the neglect of mechanisms associated with malaria dispersal related to surface water in human and vector water exposure assessment (Sinka et al., 2010; Solano‐Villarreal et al., 2019). Therefore, we aim to reassess the power of surface water in predicting malaria prevalence through replacing and supplementing these classical surface water exposure data by advanced indices of surface water.

Our research aims to address the following question: Do novel surface water exposure indices derived from high‐resolution surface water data exhibit stronger predictive power for malaria prevalence compared to precipitation? To accomplish this, we will compare the predictive performance of novel high‐resolution surface water exposure indices with various precipitation indices, considering multiple distances of influence for surface water and precipitation on malaria. Additionally, we will compare the predictive power of the novel exposure indices with commonly used simple exposure measures derived from lower‐resolution surface water products, as well as with the same novel exposures except derived from lower‐resolution surface water products.

We will achieve these objectives by assessing the predictive performance and the relative importance of malaria predictors in Boosted Regression Trees (BRT) model scenarios. In total, we model 14 scenarios, consisting of two times seven scenarios of predictors derived in either a maximum spatial context of 100 km or 10 km around the malaria observations. The seven scenarios are: (1) confounders only, (2) confounders and precipitation predictors, (3) novel surface water predictors derived from higher‐resolution products (4) novel surface water predictors derived from lower‐resolution products, (5 and 6) classic surface water predictors used in previous studies, respectively with and without precipitation and (7) a combination of precipitation and higher‐resolution novel surface water predictors.

## 2 Methods and Data

### 2.1 General Methodological Framework

**Figure 1:**
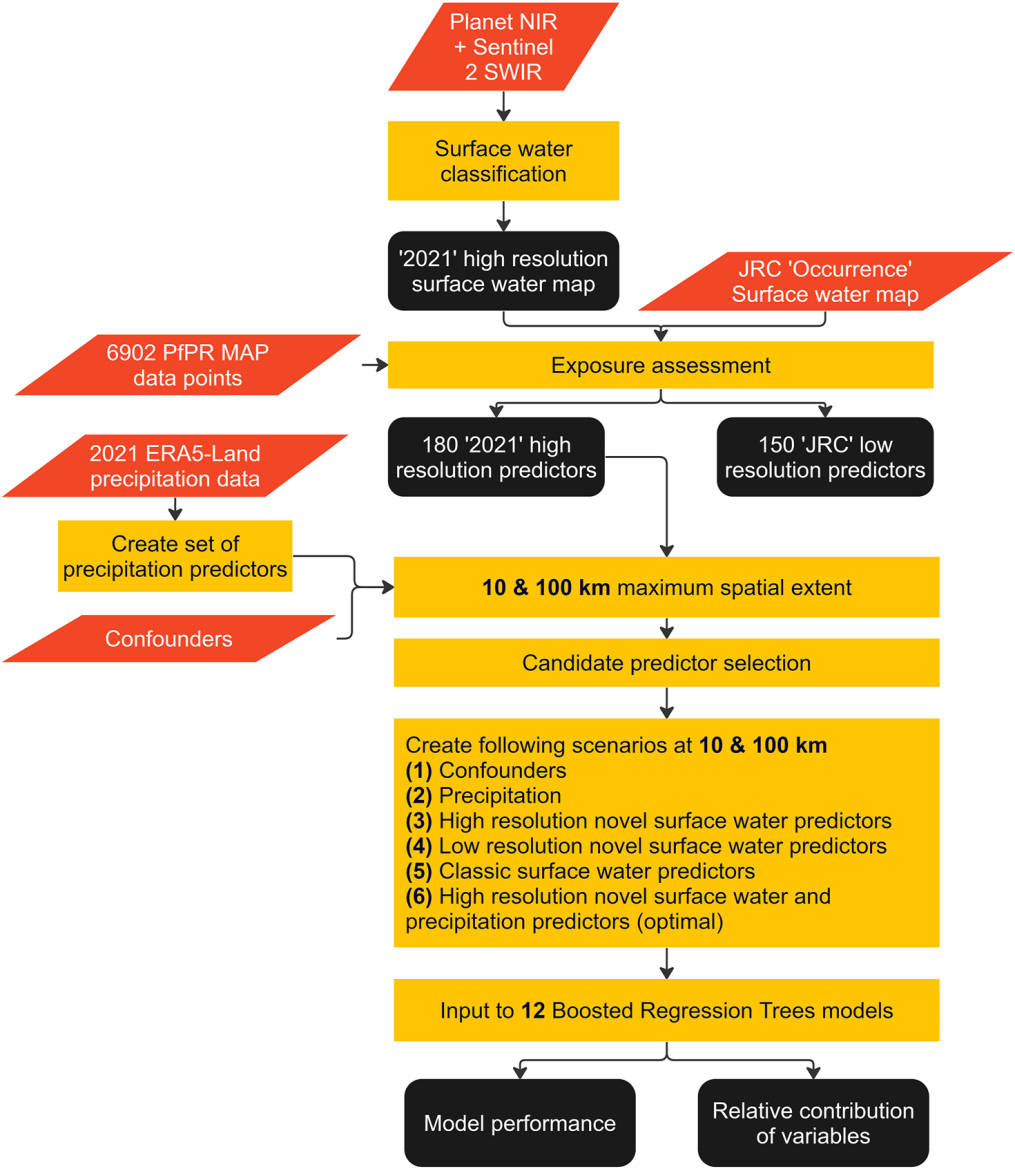
Flowchart of the workflow utilized in this study. Orange‐red diamonds are inputs, black squares are results or intermediary products and yellow boxes are operations.

For scenario (1), we incorporated four commonly used data sets containing confounding variables associated with malaria (Figure 1). In scenario (2), reanalysis precipitation predictors were derived from the ERA5‐Land Monthly Averaged—ECMWF Climate reanalysis data set (Muñoz Sabater, 2019), created similarly to Sinka et al. (2010). For scenarios (3 and 4), we required higher‐ and lower‐resolution surface water maps for extensive regions of SSA. Thus, we derived high‐resolution surface water maps for East Africa from a combination of 5 m resolution Near‐Infrared (NIR, Norway's International Climate and Forests Initiative, 2021) and 20 m resolution Sentinel‐2 Short‐wave Infrared (SWIR, Drusch et al., 2012) satellite imagery, while lower‐resolution data (30 m) was sourced from the existing Joint Research Center (JRC) surface water data set (European Commission, 2022; Pekel et al., 2016). Using these data sources, we derived novel surface water exposures representing mechanisms of malaria dispersal. These consisted of six thresholds of the minimum water persistence over time of what was considered water, five sizes of circular areas around the malaria observation point from which surface water data was extracted, three metrics that converted this water data into meaningful attributes for malaria and four minimum sizes of included waterbodies for the Average Distance to Water metric. The combination of these exposures resulted in a total of 180 high‐ and lower‐resolution candidate predictors. Next, to limit the number of predictors in scenario (3 and 4), we selected a subset of the candidate predictors that strongly associated with malaria using machine learning supported data‐analysis. For scenario (5 and 6), we derived a set of simple surface water predictors as used in Solano‐Villarreal et al. (2019), used without (5) and with (6) precipitation. For scenario (7), we combined the predictors of scenario (2) and (3). We created two variations of the scenarios by restricting the maximum spatial context for predictor derivation to 100 and 10 km, respectively. We then used the predictors of each scenario to train and validate 14 BRT models, using a data set of 6902 *Plasmodium falciparum* observations in East Africa (Hay & Snow, 2006; Moyes et al., 2022). Finally, we compared the performance and relative contribution of predictors among all models.

### 2.2 Study Area

**Figure 2:**
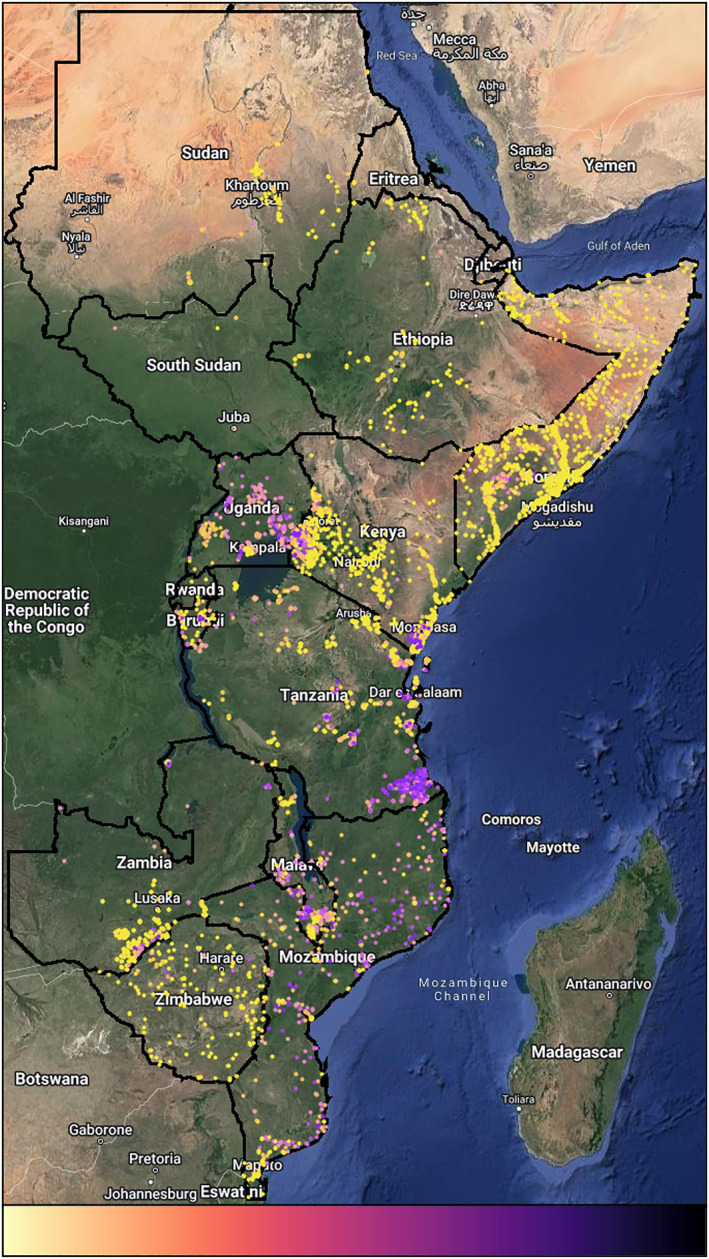
Black outlines delineate the countries included in the analysis. *P*. *falciparum* parasite rate observation locations are indicated with dots. Values range from 0 to 1, from yellow to purple, respectively.

The study area included mainland East Africa, extending from Sudan to Mozambique, covering an area of approximately 8 × 10^6^ square kilometers (Figure 2). In 2021 the area included approximately 475 million inhabitants at a median age of 18.7 years, of which 29.8% resided in urban environments (United Nations, 2022). Climates range from arid desert (BWh) in the north, savannahs (Aw) and tropical monsoons (Am) in the center and temperate (Cwb) in the south (Beck et al., 2018; Köppen, 1884).

### 2.3 Source Data Sets

To create the high resolution surface water data set, we acquired Sentinel‐2 Shortwave‐Infrared (20 m spatial and 1,610 nm spectral resolution) and Planet NICFI Basemaps' Near‐Infrared (4.77 m spatial and 846—888 nm spectral resolution) (Norway's International Climate and Forest Initiative, 2021) scenes from Google Earth Engine (Gorelick et al., 2017) for all individual months of 2021. After, we cloud corrected and aggregated the Sentinel‐2 scenes per month. The Planet scenes were pre‐processed and provided as analysis‐ready monthly mosaics by Planet.

To validate the surface water maps, we determined the Kappa Coefficient (KC) and Error of Ommission and Commission with the TOP25raster data set in the Netherlands (Section 2.4.2), as detailed concurrent surface water validation data was unavailable in East Africa. TOP25raster is a digital cartographic representation at 25 cm resolution of the TOP10NL topographical base map of the Netherlands (Kadaster, 2021). TOP10NL is made through aerial photos, panorama photos, fieldwork, and information from external sources (Kadaster, 2021). The version used in this study was updated using aerial photos collected in flights during the spring and summer of 2020.

We obtained lower resolution predictors from the European Commission's JRC Global Surface Water occurrence map v1.3 (Pekel et al., 2016), available in Earth Engine. This map, derived from Landsat 5, 7, and 8, covered a span of 32 years from 1984 to 2015 and had a resolution of 30 m. We refer to the values in this map as the 'persistence' of water pixels, representing the percentage of time they were present at specific locations throughout the acquisition period.

Precipitation predictors were derived from the ERA5‐Land Monthly Averaged—ECMWF Climate reanalysis data set (Muñoz Sabater, 2019), available in Earth Engine. The ERA5 reanalysis data set offered a comprehensive, gridded estimate of rainfall in an otherwise data sparse region at a spatial resolution of 11 km.

The Normalized Difference Water Index (NDWI) was part of both the classic benchmark and novel scenarios and was obtained from Google Earth Engine (Gorelick et al., 2017). It is calculated by Google as the NIR (845–885 nm)—SWIR (1,560–1,660 nm)/NIR + SWIR of each Landsat 8 scene at a resolution of 30 m. We then averaged these 32‐Day composites in 2021 to create the yearly mean NDWI for 2021.

We included four confounder data sets. Where possible, the acquisition date of the confounders was chosen to be close to that of the malaria observations (2003). The first confounder was the Normalized Difference Vegetation Index (NDVI), derived by Google Earth Engine. It was calculated by Google as the 32‐day average (NIR—Red)/(NIR + Red) of Landsat 7 scenes at a resolution of 30 m. We then averaged the 32‐Day composites, acquired from 2000 to 2006, to obtain an average acquisition date of 2003. The second confounder was elevation, derived by the Shuttle Radar Topography Mission, acquired in February 2000 at a resolution of 1 arc s (Farr et al., 2007), available in Earth Engine. The third was long‐term mean yearly air temperature from 1970 to 2000 at 1 km resolution from the WorldClim 2 data set (Fick & Hijmans, 2017). Lastly, there was travel time to cities at 1,000 m resolution for 2015 we used as a proxy for access to preventive and treatment interventions (Weiss et al., 2018).

We obtained malaria prevalence data for the *P*. *falciparum* parasite from the Malaria Atlas Project (Hay & Snow, 2006). The data set consisted of 6902 *P*. *falciparum* observations in East Africa, contributed by over three thousand sources, from 1984 to 2017 with an average acquisition date of 2003 (Moyes et al., 2013, 2022). Each observation refers to the proportion of positive *P*. *falciparum* tests out of the total community members tested at the site, referred to as the *P*. *falciparum* Parasite Rate (PfPR). The PfPR follows a known pattern as a function of age, rising during infancy and childhood and declining afterward as immunity develops (D. L. Smith et al., 2007). Therefore, we standardized the data so that all PfPR observation points represent the PfPR that would be present in the age range of 2–9 years old without immunization, following the Pull and Grab model by D. L. Smith et al. (2007).

### 2.4 Methods

#### 2.4.1 Derivation of the “2021” High‐Resolution Surface Water Data Set

##### 2.4.1.1 Classification Procedure

**Figure 3:**
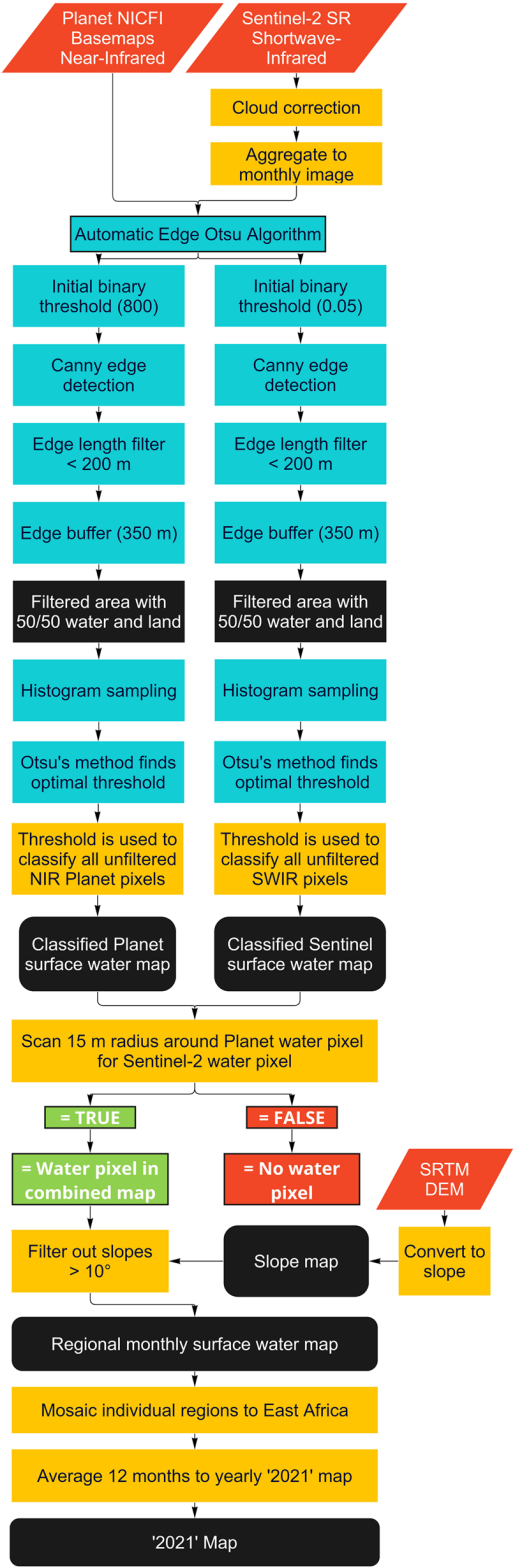
Flowchart of the creation of the “2021” map. Orange‐red diamonds are inputs, black squares are results or intermediary products, yellow boxes are operations, blue boxes are the Automatic Edge Otsu Algorithm and the green and red squares indicate conditional statements.

First, we separately classified the monthly single‐band Planet NIR and Sentinel‐2 SWIR scenes using an Automatic Otsu Thresholding algorithm by Cao et al. (2019), adapted for Earth Engine by Markert et al. (2020) (Figure 3). This algorithm first finds a threshold based on water pixel values in filtered subsections of the study area, and then uses this threshold to classify the entire unfiltered study area. It does so by a variance based automatic image thresholding technique, that finds a threshold value where the weighted variance between foreground and background pixels is least. As Otsu's method produces optimal thresholds if the image's values are bimodal, the adapted method by Cao et al. (2019) limited the region from which it obtains pixel values to an area which only contains distinct water and non‐water pixels at almost equal proportion. It first required an initial, manually set, threshold that represented an approximation of the transition between water and non‐water, which we set at 800 and 0.05 for the Planet NIR and Sentinel‐2 SWIR bands, respectively. With this threshold, the image was divided into a preliminary binary water and non‐water map, of which the transitions were detected with a Canny edge filter. To make sure that the filtered areas on which the final threshold will be made did not include landforms other than water, edges under 200 m in length were removed. Then, the remaining edge features were buffered on either side by 350 m, creating an area where, in most cases, approximately half of the values represent water, and the other half of values represent land. From the buffered area, Otsu's method sampled the pixel values and subsequently used them to find the optimal threshold. It then used this threshold to classify the entire unfiltered study area into a water and non‐water class at the resolution of the Planet NIR and Sentinel‐2 SWIR bands.

After we classified the Planet NIR and Sentinel‐2 SWIR single‐band scenes separately, we combined both to a single classified surface water map. Our algorithm scanned a radius of 15 m around each Planet pixel classified as water. If there were any SWIR pixels classified as water in that area, the pixel would remain classified as water, if not, it would become non‐water. We used this combination of two water maps from separate satellite products, because it reduces the number of false positives that are likely to be present in classifications made using single bands, as any false positives need to be present in both images to be classified as water in the combined map. Furthermore, we could increase and separately adjust the sensitivity of the thresholds of both maps, as the combined product would compensate some of the false positives that resulted from an increased sensitivity.

Some mountainous areas cast permanent shadows that were classified as water. To remove these, we first generated the slope angle from the Shuttle Radar Topography Mission digital elevation data (Farr et al., 2007), after which we removed each water pixel where the slope was above 10° degrees. We selected this value because stream gradients are typically below 10° (Church, 2002; Hack, 1973), and if they exceed this, they are fast‐flowing and generally unimportant to malaria.

To find area optimized thresholds, we created binary surface water maps separately for all African countries, except for Eritrea and Djibouti, and Uganda Burundi and Rwanda, which we grouped due to their small sizes. After, we mosaicked all separate maps and averaged the monthly East African maps to create one where each value represented the percentage of time a water pixel was present at a location. Similar to the JRC map, we refer to this value as the “persistence (%)” of water at that location. We refer to this new surface water map as the “2021” map.

#### 2.4.2 Surface Water Maps Validation

To the authors' knowledge there was no concurrent validation data in East Africa. Therefore, we validated both the high resolution surface water map and the global JRC water map in the Netherlands using the TOP25Raster (25 cm resolution) (Kadaster, 2021) as validation data. Most water in the Netherlands has little seasonal variation (Lintsen, 2002). Therefore, to obtain a binary map, we set the maps at 90% persistence. If the persistence was set lower, instead of including semi‐permanent waterbodies, it provides lenience for pixels omitted in some months of classification. Then, for both maps, we calculated the Overall Accuracy (Equation 1), KC (Stehman, 1996) and the Omission (Equation 2), and Commission (Equation 3) error. Additionally, we included a qualitative comparison between the “2021” and JRC surface water map.

(1) $$\text{Overall}\;\text{Accuracy}\;\left(\%\right)=\frac{\text{Correctly}\;\text{classified}\;\text{pixels}}{\text{Total}\;\text{number}\;\text{of}\;\text{pixels}}\;*100$$

(2) $$\text{Omission}\;\text{error}\;\left(\%\right)=\frac{\text{Incorrectly}\;\text{classified}\;\text{ground}\;\text{truth}\;\text{pixels}}{\text{Total}\;\text{number}\;\text{of}\;\text{ground}\;\text{truth}\;\text{pixels}}\;*100$$

(3) $$\text{Commission}\;\text{error}\;\left(\%\right)=\frac{\text{Incorrectly}\;\text{classified}\;\text{pixels}}{\text{Total}\;\text{number}\;\text{of}\;\text{classified}\;\text{pixels}}\;*100$$

#### 2.4.3 Derivation of Malaria Exposures

For scenarios (3, 4, and 7) we derived surface water predictors. These predictors for malaria must represent the different pathways and spatial contexts of malaria (Sinka et al., 2010; Smith et al., 2013). However, if all processes and contexts are incorporated in a model simultaneously, the total number of predictors would be exceptionally high. Yet, it is also challenging to identify a priori which processes or predictors are most important, something which is also dependent on the spatial and temporal scale (Smith et al., 2013). Therefore, instead of determining beforehand which predictors to use, first, we made a large set of 180 unique candidate predictors by combining three types of malaria relevant water exposures. These exposures were based on literature and represented pathways of how surface water may influence malaria (Smith et al., 2013). After we obtained the 180 candidate predictors (Table 1), we selected a smaller subset of high performing predictors from it (Section 2.5, scenario 3).

**Table 1:** All Exposures and How They Combine to the 180 Candidate Predictors

Predictor type	Spatial context sizes	Persistence thresholds	Minimum pixel size	Total
Number of Small Waterbodies	5	6	1	30
Percentage of Water in Spatial Context	5	6	1	30
Distance to water	5	6	4	120
Candidate predictors				180

Our general approach of the exposure assessment involved the aggregation of spatial information of surface water occurrence over an area surrounding an observation location, the so‐called spatial context. This involved the selection of the size of the spatial context, a threshold parameter for binary surface water mapping, and multiple spatial aggregation methods.

1. Size of the spatial context. The spatial context is the circular area centered at a malaria observation location assumed to influence malaria at that location. Vectors are generally limited in how far they travel from aquatic nesting sites, and although flight distances of several kilometers from nesting sites are reported, vector abundances starkly drop after 100 m from the water edge (Bomblies, 2014; Buxton et al., 2021; Ciota et al., 2012; Tuteja, 2007). Based on this, the spatial context radius should either equal the maximum fly distance of vectors or equal the area closest to water where transmission risk is highest. However, PfPR observations (Moyes et al., 2013) were denoted where testing had occurred, not where transmission had occurred. Consequently, the PfPR also depends on the whereabouts of the host. Situations with frequent human movement, such as those involving migrant workers (Aschale et al., 2018) or testing locations with a large support size such as hospitals (Moyes et al., 2013), may thus reduce the correlation between predictor and malaria incidence if only accounting for environmental factors in the proximity of the observation. Therefore, we took multiple spatial contexts instead of one, deriving predictors from very small to very large circular buffers around observations, using the following radii: 50, 100, 1, 10 and 100 km.
2. Threshold value of the surface water persistence. The value of this threshold determines what constitutes water. Vectors use permanent or semi‐permanent surface waterbodies differently for oviposition (Sinka et al., 2010; Smith et al., 2013). Thus, temporally varying waterbodies potentially have separate effects on malaria and there is no one‐size‐fits‐all definition of water/no‐water. Therefore, we varied the definition of what was surface water. The definitions included: >90% persistence, where every surface water pixel had to be present in at least 90% of the individual monthly maps until it was counted as water, >70% persistence, >50% persistence, >20% persistence, >10% persistence and lastly >0% persistence, where all observations of water pixels were included. This resulted in binary surface water occurrence maps, derived from the continuous values of the “2021” surface water maps.
3. Method for spatial aggregation of the binary surface water occurrence map. This aggregation results in an exposure index value representing a particular malaria dispersal mechanism related to surface water. We used three methods. The first exposure was the percentage of surface water within the spatial context. This represents the general availability of surface water and thus the availability of aquatic habitats for malaria transmission. However, large waterbodies have, comparatively, a much larger surface water area than smaller channels or temporary waterbodies, while the role of large waterbodies in malaria transmission is considered relatively smaller (Sinka et al., 2010; Smith et al., 2013). Therefore, as a second exposure, we counted the total number of individual waterbodies with a size below 600 m^2^ in the spatial context. The third aggregation method involved the spatial structure of waterbodies. As stated before, vectors are generally limited in how far they travel from oviposition sites (Bomblies, 2014). Both number of waterbodies and percentage of total area only captured the availability of water but not the spatial distribution or position of water with respect to land. Therefore, as the last exposure, we chose the Average Distance to Water. This metric calculated for each “2021” pixel the shortest Euclidean distance to a water pixel and then took the average of this attribute over all pixels in the spatial context. However, transmission risk gradients are reported to correspond with the proximity to trunk river alluvial plains or tributary streams (Clarke et al., 2002) and increased incidence can be related to proximity to a nearby marsh, large waterbody or other locations of flow accumulation (Smith et al., 2013). Therefore, we calculated distances to only larger patches of water pixels instead of single pixels as well. We did this through reducing the spatial resolution of the surface water maps. At 500 m, smaller waterbodies were excluded and detection started at pixels that would be detected with 500 m resolution sensors, representing medium to large rivers (Wang et al., 2010). At 1 km resolution smaller rivers were excluded and at 5 km only the largest lakes were included. This resulted in four distance maps, distance 5 m, 500 m, 1 km and 5 km surface water patch size.

The combinations of all exposures bring the total number of candidate predictors to 180 (Table 1).

### 2.5 Model Scenarios

Our research question was whether novel surface water exposure indices derived from high‐resolution surface water data can be stronger predictors of malaria prevalence compared to precipitation. Furthermore, we intended to compare the novel set of surface water exposures to the widely used simple exposure measures and an identical set of novel exposures derived from low resolution surface water products. Lastly, we want to assess the distance of influence and the relations at different scales of surface water, precipitation and malaria. To do so, we model 14 scenarios, consisting of two times seven scenarios of predictors derived in either a maximum spatial context of 100 km or 10 km around the malaria observations. The seven scenarios are: (1) confounders only, (2) confounders and precipitation predictors, (3) novel surface water predictors derived from higher‐resolution products (4) novel surface water predictors derived from lower‐resolution products, (5 and 6) classic surface water predictors used in previous studies, respectively with and without precipitation and (7) a combination of precipitation and higher‐resolution novel surface water predictors. Table 1 shows a summary of the predictor types we included in both the 10 and 100 km scenarios, and Table S1 in Supporting Information S1 shows a detailed overview of all predictors per scenario.

For the models to resemble actual malaria models, we included four commonly used confounders for malaria in all scenarios (Section 3.2). These consisted of the yearly mean NDVI, elevation (Farr et al., 2007), air temperature (Fick & Hijmans, 2017) and travel time to cities (Weiss et al., 2018) (Section 3.2.). Each confounder provided two predictors, one aggregated within a small (50 m or 1,000 m) and one within a larger spatial context (100 km or 10 km) (Table S1 in Supporting Information S1). This set of confounders remained identical between scenarios.

*Scenario* 1 (*Confounders*): Included only the set of confounders to determine the baseline predictive performance of the confounders.

*Scenario* 2 (*Precipitation*): Included precipitation, in addition to the confounders of scenario (1). Four variations of precipitation predictors, based on the precipitation predictors used by Sinka et al. (2010), were derived from the ERA5‐Land monthly data set at a resolution of 11 km (Muñoz Sabater, 2019). The average cumulative monthly rainfall in 2021, the 2021 months with the maximum and minimum cumulative precipitation and lastly the maximum absolute difference in precipitation between either the maximum or minimum precipitation month and the mean cumulative monthly precipitation in 2021, referred to as the maximum amplitude of the variation around the mean precipitation. Each variable provided two predictors, one derived within a small radius (50 m) and one within a large radius (10 or 100 km) spatial context (Table S1 in Supporting Information S1). With this selection we attempted to derive the most information from precipitation data as possible, as for surface water predictors in scenario (3) we made the same attempt.

*Scenario* 3 (*High‐resolution* | *novel*): Included, in addition to the confounders of scenario (1), a set of strong surface water predictors. To not have a much larger number of surface water predictors compared to precipitation predictors, we selected a smaller subset of the 180 candidate predictors. We evaluated the contributions and model performance of models with different subsets, where the goal was to have the highest performance with the fewest number of predictors. This amounted to a total of 16 surface water predictors, which consisted of 12 strongly performing Average Distance to Water predictors, of which seven distance maps were calculated to single pixels (5 m resolution) and five to larger aggregated water patches (S1), one Percentage of Water in Spatial Context and two Number of Small Waterbodies predictors (Table 2; Table S1 in Supporting Information S1). Additionally, we added the yearly mean NDWI at 30 m resolution (Section 2.3) due to its strong performance in the classic benchmark of scenario (5).

**Table 2:** All Scenarios and Their Included Predictor Types for the 10 and 100 km Scenarios

Scenario	Included predictors	Number of variables
(1) Confounders	All Confounders	8
	**Total**	**8**
(2) Precipitation	**Confounders** (1)	8
	All Precipitation	8
	**Total**	**16**
(3) High‐resolution | novel	**Confounders** (1)	8
	Average Distance to Water	12
	Percentage of Water in Spatial Context	1
	Number of Small Waterbodies	2
	NDWI	1
	**Total**	**24**
(4) Low‐resolution | novel	**Confounders** (1)	8
	Average Distance to Water	12
	Percentage of Water in Spatial Context	1
	NDWI	1
	**Total**	**22**
(5) Classic | No precipitation	**Confounders** (1)	8
	Shortest distance to river	1
	NDWI	1
	**Total**	**10**
(6) Classic | Precipitation	**Precipitation** (2)	16
	Shortest distance to river	1
	NDWI	1
	**Total**	**18**
(7) Optimal	**Precipitation** (2)	16
	Average Distance to Water	12
	Percentage of Water in Spatial Context	1
	Number of Small Waterbodies	2
	NDWI	1
	**Total**	**32**

*Note*. Bolded names equate all predictors of that scenario.

*Scenario* 4 (*Low‐resolution | novel*): Included the same predictors as scenario (3), except that the seven high‐resolution predictors were now derived from the lower resolution JRC surface water map (30 m spatial resolution). As the condition of small waterbodies predictor was that the bodies were below 600 m^2^ and one JRC pixel is 900 m^2^, we omitted it in this scenario.

*Scenario 5* (*Classic surface water predictors* | *No precipitation*): Instead of the Novel surface water predictors in (3 and 4), we included a set of classic surface water predictors identical to those used in a malaria prediction study by Solano‐Villarreal et al. (2019). The first was the yearly mean NDWI at 30 m resolution (Section 3.2.2). The second was calculated as the shortest Euclidean distance to the nearest water using the JRC maps at 50% persistence. Solano‐Villarreal et al. (2019) chose this persistence level to include main and secondary rivers.

*Scenario* 6 (*Classic surface water predictors*): Included the precipitation predictors in addition to the classic surface water predictors of Solano‐Villarreal et al. (2019), similar to a classic model set‐up seen in previous studies.

*Scenario* 7 (*Optimal combined*): scenario including all predictors from the precipitation scenario (2), in addition to the surface water predictors of scenario (3).

### 2.6 Boosted Regression Tree Analysis

BRT models (Elith et al., 2008) were created using the “gbm” (Greenwel et al., 2022) and “dismo” (Hijmans et al., 2022) packages in R (R Core Team, 2021). For each scenario the 6902 PfPR observations were divided into a training (90%) and validation (10%) data set. We used 10‐fold cross‐validation through the “gbm.step” function in combination with the rules of thumb of Elith et al. (2008) to find the combinations of parameters and numbers of trees with the lowest deviance. We found that slightly different combinations produced only very slightly different optimal deviance between all BRT models. Thus, we decided to set the optimal learning rate (0.005), tree complexity (10), bag fraction (0.6) and number of trees (5,000) the same for all scenarios.

To eliminate the effect of the composition of the training data set, we ran and validated each model 50 times, each with a different randomized set of training (90%) and validation (10%) data. The adjusted *R* ^2^, We derived the Residual deviance and RMSE for each run to indicate model performance. Furthermore, we supplemented all models with the average Relative Contribution over 50 runs of each variable category. The Relative Contribution is determined by how often a variable is selected for splits, weighted by the squared model improvement resulting from consecutive splits (Elith et al., 2008; Solano‐Villarreal et al., 2019).

## 3 Results

### 3.1 “2021” and JRC Surface Water Map Accuracies

Validated against the 25 cm resolution TOP25Raster map, the Errors of Commission of the “2021” workflow and JRC map were similar. However, the classified NIR + SWIR method had a substantially lower Error of Omission (Table 3).

**Table 3:** Validation of Different Map Products Against the 25 cm Resolution TOP25Raster Map in the Netherlands

Classification inputs used	Overall accuracy (%)	Error of commission (water) (%)	Error of omission (water) (%)	Kappa coefficient (−)
Single‐band Planet NIR	94.5%	9.9%	12.8%	0.850
Single‐band Sentinel‐2 SWIR	94.4%	9.8%	12.2%	0.853
Combined NIR + SWIR	96.1%	2.6%	13.2%	0.891
JRC	94.2%	1.7%	19.9%	0.847

*Note*. Single‐band NIR and Sentinel‐2 SWIR indicate the separately classified maps. Combined NIR + SWIR was the final product. JRC indicates the JRC map at 90% persistence in the Netherlands.

**Figure 4:**
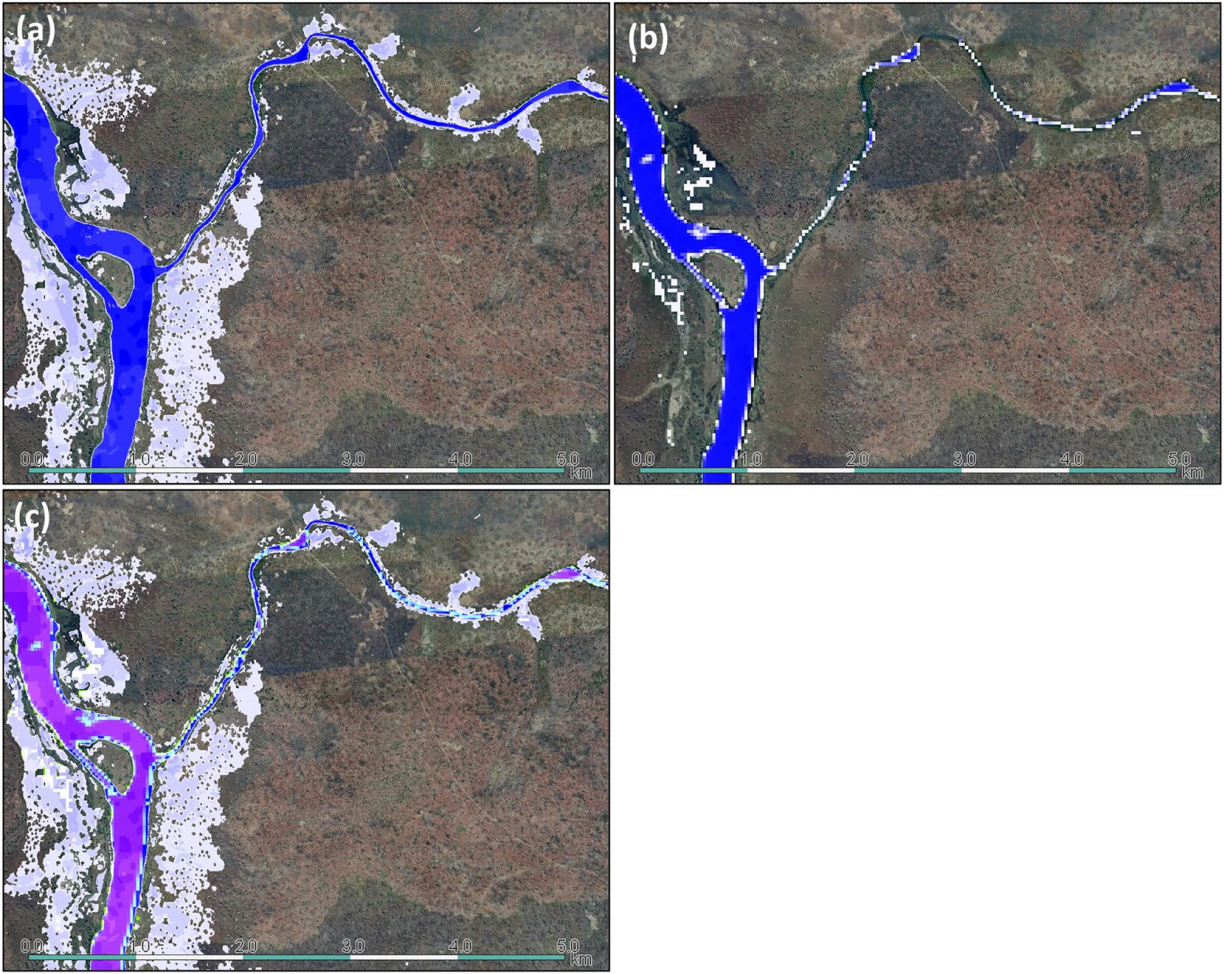
A comparison between the “2021” surface water map (a, 5 m resolution) and the Joint Research Center (JRC) surface water map (b, 30 m resolution). Full blue color indicates water was present 100% of the recorded period, values with no water are masked, values approaching 0% persistence are white. (c) Shows the combination of both, where only JRC = Green, only “2021” = dark blue, if both maps have values = dark purple to light blue (darker purples meaning both maps have high persistence, light purple = JRC map higher persistence, light blue = “2021” map higher persistence). Background is satellite imagery from Google. Location is near Chembe, at the border of Zambia and Congo‐Kinshasa (11°59′25.9″S 28°46′00.1″E).

In general, the JRC surface water map captures larger rivers seen in the Google Earth Engine satellite imagery relatively similar to the “2021” map, however, it fails to capture the smaller tributaries which the high‐resolution maps do accurately classify (Figures 4a–4c).

### 3.2 Scenario Performance and Predictor Contribution

**Figure 5:**
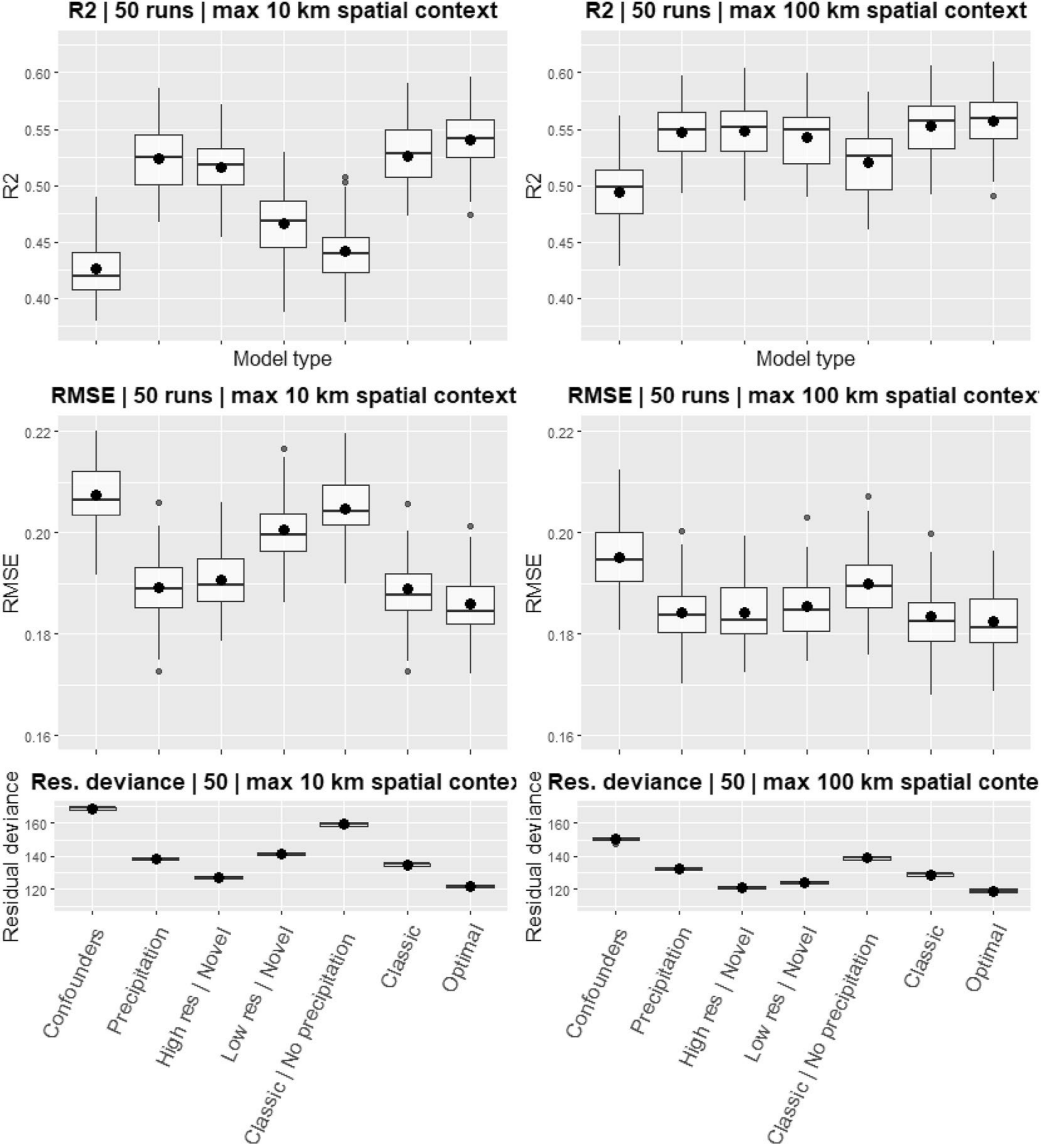
*R* ^2^, RMSE and Residual deviance of both the smaller and larger radius spatial contexts of the seven scenarios as described in Table 1. Big dots indicate the mean value over 50 runs; bold lines the median; boxes, the 25th and 75th percentile; vertical lines the range of values; small dots data points outside 1.5 time s the interquartile range from the upper or lower quartile.

**Figure 6:**
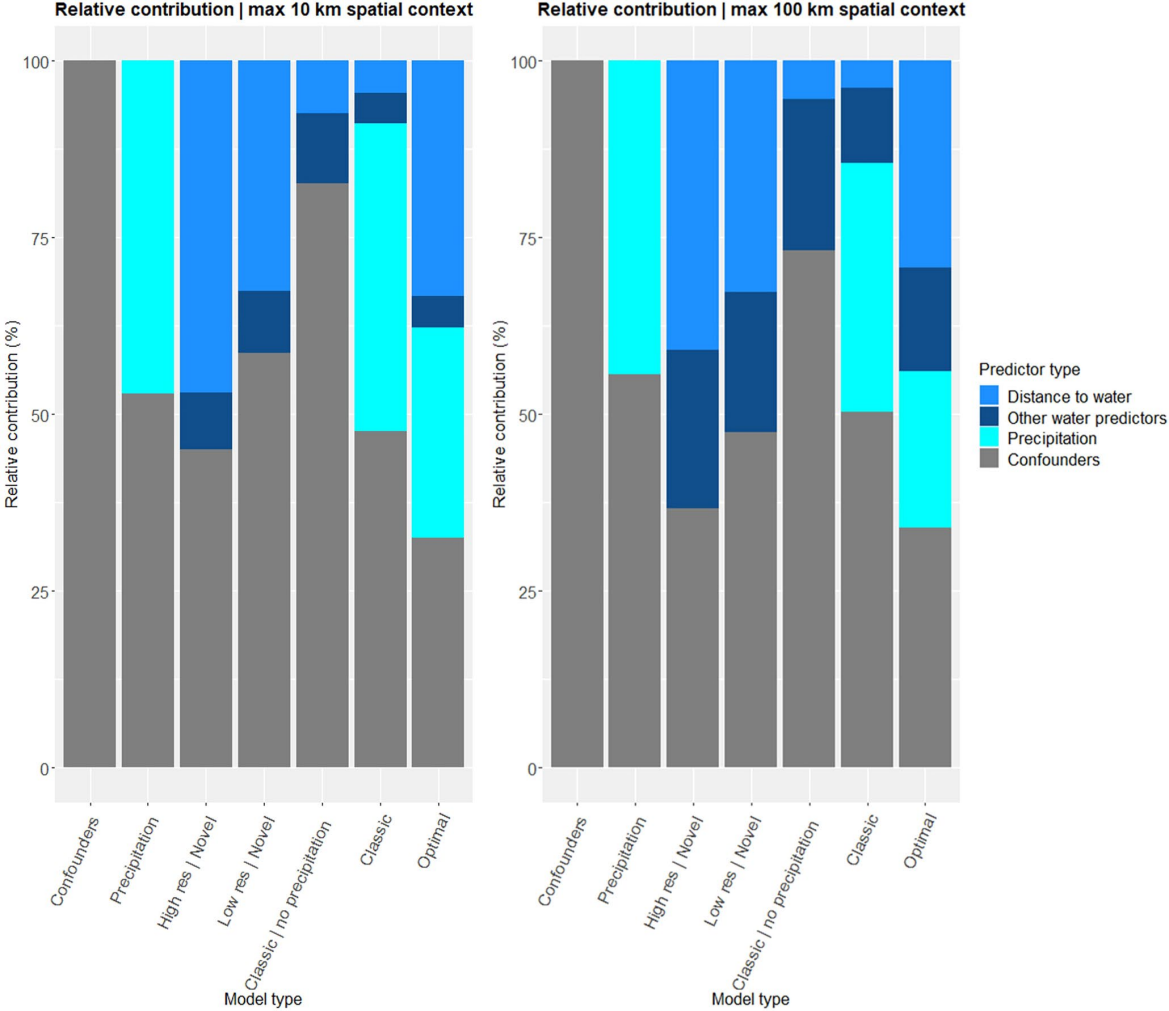
The total Relative Contributions of predictor categories to model performance. Distance to water category consisted of shortest distance to water in the “Classic” scenario and average distance to water in the other scenarios. The “Other water predictors” consist of Normalized Difference Water Index, Percentage of Water in Spatial Context and Number of Small Waterbodies predictors. Table 1 and Table S1 in Supporting Information S1 provide a full overview of the predictors in each scenario.

In the maximum 10 km spatial context variation, the scenario with High‐resolution Novel surface water predictors (3) had an almost equal *R* ^2^ compared to the Only Precipitation predictors scenario (2, Figure 6). Furthermore, the *R* ^2^ of the Optimal scenario (7) exceeded that of the Classic scenario (6). Lastly, the Low‐resolution Novel surface water predictors scenario (4) had a substantially lower *R* ^2^ compared to the High‐resolution Novel surface water scenario (3), however, the Classic scenario without precipitation (5) had the lowest *R* ^2^ of all non‐confounders scenarios. The residual deviance and RMSE (Figure 5) followed the same patterns.

In the maximum 100 km spatial context variation, the scenario with Only High‐resolution Novel surface water predictors (3) had a slightly higher *R* ^2^ compared to the Only Precipitation predictors scenario (2). Aside from this, largely similar patterns to the 10 km variation are seen. However, the overall *R* ^2^ is higher, while the variation in *R* ^2^ between scenarios is substantially smaller. Only the Only Confounders (1) and Classic without precipitation (5) scenarios are noticeably lower compared to the other scenarios. Nonetheless, this difference was still less compared to that seen in the maximum 10 km variation.

In the maximum 10 km spatial context variation, the Relative Contribution of Distance to water predictors was considerably higher in the Optimal scenario (7) compared to the Classic scenario (6, Figure 6). Furthermore, while the Relative Contribution of water related predictors was substantially smaller in comparison to precipitation in the Classic scenario (6), it exceeded that of precipitation in the Optimal scenario (7).

Compared to the 10 km variation, the main difference in the 100 km variation is that the contribution of NDWI, Percentage of Water in Spatial Context and Number of Small Waterbodies predictors have increased substantially in all scenarios (Figure 6). This increase is mainly due to the higher Relative Contribution of the NDWI predictor.

## 4 Discussion

Our research aimed to address whether novel surface water exposure indices derived from high‐resolution surface water data can be stronger predictors of malaria prevalence compared to precipitation. Therefore, we compared the predictive power of novel high‐resolution surface water exposure indices with that of various precipitation indices, considering multiple distances of influence of surface water and precipitation on malaria. Also, we compared the predictive power for malaria prevalence of novel exposure indices derived from high‐resolution products with identical exposures derived from lower‐resolution surface water products, and widely used simple exposure measures.

### 4.1 Accuracy of the “2021” and JRC Maps

The accuracy in the Netherlands of the “2021” surface water map derived here was higher compared to the JRC map, lowering the Error of Omission (6.7%) by a factor equal to seven times the increase of the Error of Commission (0.9%). However, we had no validation in East Africa. Thus, although area optimized thresholds were used, the accuracy of our workflow may vary between our region and the different regions in East‐Africa. In the case that the accuracies are reversed in East‐Africa, the higher performance of the high‐resolution scenario may have other origins. However, Figure 4 Shows that the “2021” map classified waterbodies more accurately at that location, a pattern which is visible in many other locations as well (Kalthof et al., 2023). Therefore, we do not believe that it is likely that the accuracies of the JRC map are higher in East‐Africa. A more extensive analysis on the useability of high‐resolution Planet data for surface water mapping is a topic for future research, considering it is one of the few high‐resolution openly available data sets

### 4.2 Surface Water Versus Precipitation Predictors

High‐resolution surface water derived indices performed similar to precipitation indices in both the 10 and 100 km spatial context variations. Furthermore, the Relative Contribution of the surface water derived indices exceeded those of precipitation in both the 10 and 100 km variation of the “Optimal” scenario (7). Therefore, it appears that at this resolution, novel surface water predictors can perform as good as or outperform precipitation predictors in both spatial contexts.

This contrasts the performance of surface water predictors derived from lower‐resolution products, which do appear to be spatial context dependent. In the 100 km variation, the lower‐resolution surface water predictor model (scenario 4) performed similarly to precipitation (scenario 3), but in the 10 km variation it performed substantially worse. The poor performance at small scales could be explained by the fact that, at small to intermediate scale, proximity to small waterbodies and trunk river alluvial plains or tributary streams is most important for malaria transmission, as these contain oviposition sites (Clarke et al., 2002; Minakawa et al., 2005; Sinka et al., 2010; Smith et al., 2013). However, the lower‐resolution JRC map is unable to capture such smaller waterbodies (McMahon et al., 2021; Mushinzimana et al., 2006), whereas the validation of the “2021” maps presented here indicates that our higher resolution maps are able to capture these. In contrast, at large scales, climatically determined surface water patterns are the most important determinants for malaria transmission (Smith et al., 2013). Furthermore, at this scale, the difference in resolution does not strongly affect the classification of surface water, and the deviation between the JRC and “2021” maps is relatively small. This suggests that at smaller spatial contexts, high‐resolution data is required for accurate prediction, whereas at larger spatial contexts the importance of high resolution is lower. Future research could analyze spatial contexts below 10 km in combination with higher resolution surface water data, to determine if the required resolution for accurate predictive performance further increases at smaller scales. Additionally, the role of resolution and extent in other malaria exposures that are more variable over shorter distances than the resolution of the currently used data, such as vegetation or temperature, needs to be studied.

When comparing our results to similar studies that utilize exposures in BRT models for predicting malaria occurrence, both at large (Sinka et al., 2010) and small (McMahon et al., 2021; Solano‐Villarreal et al., 2019) spatial contexts, we observe partial correspondence. These studies used lower‐resolution surface water maps and reported low importance of surface water predictors. However, the reported low importance persists across both small and large spatial contexts, indicating factors that are unaccounted for. We suggest that this factor is our use of novel exposure indices. This is corroborated by the performance of the only novel surface water exposures scenarios (3, 4) compared to that of the only classic surface water predictor scenario (5), where in both spatial context variations scenario (5) performs substantially worse. Additionally, the “Optimal” (7) outperforms the “Classic” (6) scenario, which has less sophisticated surface water indices.

Both “Classic” scenarios (5, 6) followed a pattern reported in similar studies, where surface water predictors contribute relatively little to model performance (McMahon et al., 2021; Sinka et al., 2010; Solano‐Villarreal et al., 2019). As the contribution of the NDWI, Percentage of Water in Spatial Context and Number of Small Waterbodies predictor group is similar between the “Optimal” and “Classic” model scenario, the greater Relative Contribution and better model performance could be attributed to the inclusion of the Average Distance to Water predictors instead of the Shortest Distance to River predictor. The Average Distance to Water predictors may thus contain additional surface water information important for malaria transmission that is not captured by the Shortest Distance to River predictor. If settlements sprawl or if there is frequent human movement between villages (McMahon et al., 2021), a short distance to river at the observation location may not be representative of the dispersal mechanisms at all possible locations of transmission. In contrast, Average Distance to Water represents the general location of water apropos land and the connectivity of waterbodies throughout the set spatial context, and by using multiple sizes of the spatial context and different persistencies of water this is done for more than one spatial context and waterbody temporality. Furthermore, taking only rivers into account ignores both the smaller and larger scale surface water determinants of malaria transmission (Smith et al., 2013). Small and temporary bodies are moved past, whereas large bodies are omitted as the Shortest Distance to River predictor ignores any body of water beyond the first and closest with at least 50% persistence. We suggest that because the Average Distance to Water predictor does incorporate these processes, scenarios with the novel exposure indices have a higher predictive power for malaria compared to the scenarios with classically used simple predictors.

### 4.3 10 Versus 100 km Spatial Context Variations

As a whole, the 100 km scenarios outperformed the 10 km scenarios. This contrasts the study of Brock et al. (2019), who found that predictors derived in smaller spatial contexts are more relevant to disease occurrence, which is expected based on vector behavior (Smith et al., 2013). We propose two factors that contribute to this. (a) We removed the temporal component of the malaria observations, because the Malaria Atlas Project PfPR observations (1970–2016) and surface water and precipitation predictors (2021) were not concurrent. Thus, as local surface water conditions are more variable over time compared to average conditions of a larger area, larger spatial contexts may better capture malaria patterns. (b) PfPR depends on the whereabouts of the host, thus in situations with frequent human movement, such as those involving migrant workers (Aschale et al., 2018) or testing locations with a large support size such as hospitals (Moyes et al., 2013), the relation of predictors derived in large spatial contexts and malaria may be stronger. Future studies may analyze whether these patterns still occur when using concurrent surface water and PfPR observations that account for host movement.

## 5 Conclusion

This study aimed to assess the predictive power of novel exposure indices derived from high‐resolution surface water data in comparison to precipitation for predicting malaria prevalence. Surface water predictors can be equally strong or better predictors for malaria compared to precipitation. However, for this, in smaller spatial contexts, it is required for the indices to be derived from sufficiently high‐resolution maps. Additionally, at both small and large spatial contexts, the predictors need to resemble the processes of malaria dispersion closely to rival the performance of precipitation predictors. The findings highlight the importance of developing similar high‐resolution surface water maps in malaria‐endemic regions. Moreover, the study emphasizes the need to design superior predictors that leverage the benefits of high‐resolution data, ultimately enhancing malaria prediction and aiding efforts to combat its spread. In conclusion, this research underscores the potential of high‐resolution surface water data and associated indices as valuable tools in malaria research and intervention strategies. Therefore, continued advancements in mapping techniques and predictive models are needed to contribute to improved understanding and control of malaria transmission dynamics.

## Conflict of Interest

The authors declare no conflicts of interest relevant to this study.

## Supporting information

Table S1

## Data Availability

Planet basemaps (Norway's International Climate and Forest Initiative, 2021) and Sentinel‐2 (Drusch et al., 2012) satellite scenes were used for the “2021” map. The JRC surface water map (Pekel et al., 2016), and the TOP10NL (Kadaster, 2021) were used to validate and compare the “2021” map. The ERA5‐Land (Muñoz Sabater, 2019), NDVI and NDWI were obtained from Google Earth Engine (Gorelick et al., 2017), and the WorldClim 2 data set from Fick and Hijmans (2017). Travel time to cities and the PfPR observations were obtained from the Malaria Atlas Project data viewer (Hay & Snow, 2006; Malaria Atlas Project, 2022; Moyes et al., 2022). BRT models (Elith et al., 2008) were created using the “gbm” (Greenwel et al., 2022) and “dismo” (Hijmans et al., 2022) packages in R (R Core Team, 2021). All scripts and data, including the “2021” map are available through Zenodo (Kalthof et al., 2023).
