# Supplementary material for: Predicting Continental Scale Malaria With Land Surface Water Predictors Based on Malaria Dispersal Mechanisms and High‐Resolution Earth Observation Data
Source: Geohealth. 2023 Oct 10;7(10):e2023GH000811. doi: 10.1029/2023GH000811 (PMC10564405; doi:10.1029/2023GH000811)
Supplement: Supplementary file 1 — Table S1 [file GH2-7-e2023GH000811-s001.pdf]

AGU GeoHealth

Supporting Information for

Predicting continental scale malaria with land surface water predictors based on malaria dispersal mechanisms and high-resolution earth observation data

Maurice W.M.L. Kalthof [1], Mathieu Gravey[2], Flore Wijnands[3] and Derek Karssenberg[4]

[1]Institute for Environmental Studies (IVM), Vrije Universiteit Amsterdam, Amsterdam, Netherlands.

[2] Institute for Interdisciplinary Mountain Research, Österreichische Akademie der Wissenschaften, Innsbruck, Austria

[3] Institutionen för Geologiska Vetenskaper, Stockholm University, Stockholm, Sweden

[4] Department of Physical Geography, Utrecht University, Utrecht, the Netherlands

Contents of this file

Table S1

Introduction

This supporting information contains the exact variables used in each of the 14 models used in the main paper. The format of the descriptive names are described in the table caption.

10 km Variation				
**Scenario**	**Confounders**	**Precipitation**	**Distance to water**	**NDWI v PCTWA v NRWABO**
Confounders (1)	NDVI_50<br>NDVI_b10000<br>Air_temperature_b1000<br>Air_temperature_b10000<br>Elevation_b10000<br>Elevation_b50<br>Travel_time_to_cities_b10000<br>Travel_time_to_cities_b1000	-	-	-
Precipitation (2)	**Confounders (1)**	Max_precipitation_month_b50<br>Max_precipitation_month_b10000<br>Min_precipitation_month_b50<br>Min_precipitation_month_b10000<br>Max_amplitude_precipitation_b50<br>Max_amplitude_precipitation_b10000<br>Average_precipitation_b10000<br>Average_precipitation_b50	-	-
High res | Novel (3)	**Confounders (1)**	-	Average_distance_to_water_b50p50<br>Average_distance_to_water_b1000p0<br>Average_distance_to_water_b1000p10<br>Average_distance_to_water_b1000p70<br>Average_distance_to_water_b10000p0<br>Average_distance_to_water_b10000p10<br>Average_distance_to_water_b10000p70<br>Average_distance_to_water500_b50p10<br>Average_distance_to_water1000_b10000p70<br>Average_distance_to_water1000_b10000p10<br>Average_distance_to_water5000_b10000p90	Nr_of_waterbodies_b1000p10<br>Nr_of_waterbodies_b10000p10<br>Percentage_water_area_b10000p90<br>NDWI_b10000

			Average_distance_to_water5000_b10000p70	
Low res | Novel (4)	**Confounders (1)**	-	Average_distance_to_water30_b50p50<br>Average_distance_to_water30_b1000p0<br>Average_distance_to_water30_b1000p10<br>Average_distance_to_water30_b1000p70<br>Average_distance_to_water30_b10000p0<br>Average_distance_to_water30_b10000p10<br>Average_distance_to_water30_b10000p70<br>Average_distance_to_water500_b50p10<br>Average_distance_to_water1000_b10000p10<br>Average_distance_to_water1000_b10000p70<br>Average_distance_to_water5000_b10000p90<br>Average_distance_to_water5000_b10000p70	Percentage_water_area30_b10000p90<br>NDWI_b10000
Classic | No precipitation (5)	**Confounders (1)**		Shortest_distance_to_water30_p50	NDWI_b10000
Classic (6)	**Confounders (1)**	**Precipitation (2)**	Shortest_distance_to_water30_p50	NDWI_b10000
Optimal (7)	**Confounders (1)**	**Precipitation (2)**	**High res | Novel – Distance to water (3)**	**High res | Novel - NDWI v PCTWA v NRWABO (3)**
**100 km variation**				
**Scenario**	**Confounders**	**Precipitation**	**Distance to water**	**NDWI v PCTWA v NRWABO**
Confounders (1)	NDVI_b50<br>NDVI_b100000<br>Air_temperature _b1000<br>Air_temperature _b100000<br>Elevation _b100000<br>Elevation_b50<br>Travel_time_to_cities _b100000<br>Travel_time_to_cities _b1000	-	-	-
Precipitation (2)	**Confounders (1)**	Max_precipitation_month _b50<br>Max_precipitation_month _b100000<br>Min_precipitation_month _b50<br>Min_precipitation_month _b100000	-	-

		Max_amplitude_preci pitation _b50<br>Max_amplitude_preci pitation _b10000<br>Average_precipitation _b100000<br>Average_precipitation _b50		
High res | Novel (3)	**Confounders (1)**	-	Average_distance_to_wate r _b50p50<br>Average_distance_to_wate r _b1000p70<br>Average_distance_to_wate r _b10000p0<br>Average_distance_to_wate r _b10000p10<br>Average_distance_to_wate r _b100000p0<br>Average_distance_to_wate r _b100000p10<br>Average_distance_to_wate r _b100000p70<br>Average_distance_to_wate r500_b50p10<br>Average_distance_to_wate r1000_b10000p10<br>Average_distance_to_wate r1000_b100000p70<br>Average_distance_to_wate r5000_b100000p70<br>Average_distance_to_wate r5000_b100000p90	Nr_of_waterbodies_b1 0000p10<br>Nr_of_waterbodies_b1 00000p10<br>Percentage_water_area _b100000p90<br>waterNDWI_b100000
Low res | Novel (4)	**Confounders (1)**	-	Average_distance_to_wate r30_b50p50<br>Average_distance_to_wate r30_b1000p70<br>Average_distance_to_wate r30_b10000p0<br>Average_distance_to_wate r30_b10000p10<br>Average_distance_to_wate r30_b100000p0<br>Average_distance_to_wate r30_b100000p10<br>Average_distance_to_wate r30_b100000p70<br>Average_distance_to_wate r500_b50p10<br>Average_distance_to_wate r1000_b10000p10<br>Average_distance_to_wate r1000_b100000p70<br>Average_distance_to_wate rr5000_b100000p70<br>Average_distance_to_wate r5000_b100000p90	NDWI_b100000<br>Percentage_water_area _b100000p90

Classic | No precipitation (5)	**Confounders (1)**		Shortest_distance_to_water30_p50	NDWI_b100000
Classic (6)	**Confounders (1)**	**Precipitation (2)**	Shortest_distance_to_water30_p50	NDWI_b100000
Optimal (7)	**Confounders (1)**	**Precipitation (2)**	**High res | Novel – Distance to water (3)**	**High res | Novel - NDWI v PCTWA v NRWABO (4)**

Table S1. Variables included in the 10 and 100 km radius spatial context scenarios . _bX means derived in a buffer/spatial context of X m, pX means derived from a binary surface water map where the persistence was set as >X %, the X in average_distance_to_waterX indicates that it was derived from a surface water map with X m resolution instead of 5 m resolution. Columns are predictor types, rows are all predictors in that scenario. Bold equates all predictors of that scenario.
